# Effects of Growth Hormone and Pioglitazone in Viscerally Obese Adults with Impaired Glucose Tolerance: A Factorial Clinical Trial

**DOI:** 10.1371/journal.pctr.0020021

**Published:** 2007-05-04

**Authors:** Hamdee Attallah, Anne L Friedlander, Matilde Nino-Murcia, Andrew R Hoffman

**Affiliations:** 1 Department of Medicine, Wayne State University, Detroit, Michigan, United States of America; 2 Geriatric Research Education and Clinical Center, Veterans Affairs Palo Alto Health Care System, Palo Alto, California, United States of America; 3 Department of Radiology, Veterans Affairs Palo Alto Health Care System, Palo Alto, California, United States of America; 4 Department of Medicine, Stanford University, Stanford, California, United States of America

## Abstract

**Objective:**

Recombinant human growth hormone (GH) and pioglitazone (PIO) in abdominally obese adults with impaired glucose tolerance were evaluated under the hypothesis that the combination attenuates GH-induced increases in glucose concentrations, reduces visceral adipose tissue (VAT), and improves insulin sensitivity over time.

**Design:**

Randomized, double-blind, placebo-controlled, 2 × 2 factorial design.

**Setting:**

Veterans Affairs Palo Alto Health Care System, Palo Alto, California, United States.

**Participants:**

62 abdominally obese adults aged 40–75 with impaired glucose tolerance.

**Interventions:**

GH (8 μg/kg/d, or placebo) and pioglitazone (30 mg/d, or placebo) for 40 wk.

**Outcome Measures:**

Baseline and after 40 wk of treatment, VAT content was quantified by CT scan, glucose tolerance was assessed using a 75-g oral glucose tolerance test, and insulin sensitivity was measured using steady-state plasma glucose levels obtained during insulin suppression test.

**Results:**

Baseline: body mass index (BMI), plasma glucose, and visceral fat content were similar. 40 wk: visceral fat area declined 23.9 ± 7.4 cm^2^ in GH group, mean difference from placebo: −28.1 cm^2^ (95% CI −49.9 to −6.3 cm^2^; *p* = 0.02). Insulin resistance declined 52 ± 11.8 mg/dl with PIO, mean difference from placebo of −58.8 mg/dl (95% CI −99.7 to −18.0 mg/dl; *p* = 0.01). VAT and SSPG declined with GH and PIO combined, mean differences from placebo of −31.4 cm^2^ (95% CI −56.5 cm^2^ to −6.3 cm^2^; *p* = 0.02) and −55.3 mg/dl (95% CI −103.9 to −6.7 mg/dl; *p* = 0.02), respectively. Fasting plasma glucose increased transiently in GH group. No significant changes in BMI were observed.

**Conclusions:**

Addition of PIO to GH attenuated the short-term diabetogenic effect of GH; the drug combination reduced VAT and insulin resistance over time. GH plus PIO may have added benefit on body composition and insulin sensitivity in the metabolic syndrome.

## Editorial Commentary

**Background:** People who are overweight are at higher risk of developing type 2 diabetes, particularly if they have impaired glucose tolerance (IGT). When an individual has IGT, their cells are not able to respond properly to insulin in the blood, which means that blood sugar levels can remain high, and fat cells do not take up fatty acids from blood at the rate they should. The term prediabetes is often used to refer to these linked characteristics. However, if such individuals are able to lose weight they can reduce their chances of becoming diabetic in the future. In particular, loss of a particular type of fat, the visceral fat (packed in around the internal organs, as opposed to fat immediately under the skin), is thought to be beneficial for people at risk of developing type 2 diabetes. Some researchers have suggested that giving human growth hormone (GH) to people who are overweight might help reduce their levels of visceral fat. At the same time, drugs known as thiazolidinediones are currently used, in combination with other drugs, diet, and exercise, as a treatment for type 2 diabetes. The researchers carrying out this study wanted to find out whether combining treatment with human GH and a thiazolidinedione, pioglitazone (PIO), would reduce levels of visceral fat and improve glucose metabolism in overweight adults with IGT. The researchers specifically planned to compare the changes in these primary outcomes amongst people receiving both human GH and PIO for 40 weeks with the changes in individuals receiving placebo only; additional comparisons were also done for individuals receiving either drug alone, as compared to placebo.

**What this trial shows:** A total of 76 participants were randomized and received the treatment allocated to them, but only 62 participants were included in the final analyses due to losses to follow-up. The primary outcomes being compared at baseline and after 40 weeks of treatment were the change in visceral fat levels and change in individuals' sensitivity to insulin. Individuals receiving GH experienced a drop in visceral fat area over the 40 weeks of the trial, as compared to placebo, whilst PIO alone did not seem to have an effect on visceral fat area. Individuals receiving both GH and PIO, however, also showed a decrease in visceral fat area. When examining the effect on insulin resistance, GH alone did not seem to have an effect on the ability to respond to insulin. However, administration of PIO alone did bring about a decrease in insulin resistance levels, as compared to placebo, and individuals receiving both GH and PIO together also experienced a drop in insulin resistance. The trial was not designed to detect statistically significant differences in side effects between the groups studied, but some side effects, such as build-up of fluid in the limbs and joint stiffness, seemed to be more common in the groups receiving drug treatment than in the placebo group.

**Strengths and limitations:** Although the trial was small, enough participants were recruited to detect statistically significant changes in the primary outcomes. Strengths of the trial include the use of appropriate techniques to conceal the randomization sequence from investigators recruiting participants into the trial and blinding of both participants and investigators to the treatments that an individual would receive. However, one limitation includes the fact that the likelihood of developing diabetes was not directly measured as an outcome in this trial, and it is therefore not possible to conclude from these results that administration of GH, PIO, or both combined, will help prevent diabetes amongst overweight people with IGT. Finally, this trial compared the drug interventions directly with placebo and not with behavioral interventions such as diet and exercise, which are normally recommended for the prevention of diabetes amongst overweight people. It would be important to further investigate the efficacy, harms, and costs of these drugs directly against nondrug interventions before making any recommendations about their clinical use.

**Contribution to the evidence:** Other studies have shown that PIO administration has beneficial effects on insulin sensitivity in people with type 2 diabetes. This study adds evidence confirming that PIO is likely to have similar effects in people who are not diabetic but who are overweight and who have IGT. The study also adds data regarding the effect of PIO and GH combined in such populations; giving both drugs together seemed to have beneficial effects on visceral fat area and insulin sensitivity, as compared to placebo.

## INTRODUCTION

Overweight adults with impaired glucose tolerance (IGT) have a 5%–10% risk of developing diabetes per year, and insulin resistance in the context of inadequate beta cell compensation is an important cause of progression to diabetes [1]. Weight loss has been shown to improve insulin sensitivity and prevent or delay progression to diabetes [2–5]. According to recent studies, the improvement in insulin sensitivity that occurs with weight loss is closely linked to the reduction of visceral adipose tissue (VAT), the collection of intra-abdominal adipose depots that includes omental and intrahepatic fat [6–7]. After controlling for body mass index (BMI), whole body and subcutaneous fat, only VAT is an independent predictor of endogenous insulin sensitivity and glucose metabolism before or after weight loss [8–10]. This, in turn, suggests that reducing visceral fat is crucial to improving insulin sensitivity and preventing diabetes in high-risk individuals.

Recombinant human growth hormone (GH) is a lipolytic drug that reduces total body, abdominal, and visceral fat in GH-deficient adults [11–13]. Several studies have reported as much as 25%–45% reductions in VAT following GH replacement in this population [14–16]. Like GH deficient adults, abdominally obese individuals have increased VAT and insulin resistance, and continuous 24-hour measurements indicate that GH levels are below normal [17]. Recent studies suggest that GH improves insulin sensitivity when administered over time in men with large waist circumferences (WCs), and this improvement has been indirectly attributed to visceral fat reduction [18–21]. The potential benefit of long-term GH administration on insulin sensitivity contrasts with our traditional understanding of direct GH-induced antagonism of insulin action, particularly during acute treatment [13,22]. While GH has been extensively studied in adults who were normoglycemic at baseline, less is known about the short- and long-term effects of GH in adults with IGT or diabetes.

In obese type 2 diabetics, insulin-sensitizing drugs known as thiazolidinediones (TZDs) also have been reported to reduce visceral fat and improve insulin sensitivity [23]. The combination of a TZD and GH in rodents was shown to counter the short-term, transient diabetogenic effect of GH and reduce both visceral adiposity and insulin resistance over time [24–25]. Similar studies assessing the combined use of a TZD and GH in humans have not been performed.

The purpose of this study was to determine the effects of GH and the TZD pioglitazone (PIO) alone and in combination on glucose metabolism, visceral adiposity, and insulin sensitivity in abdominally obese adults with IGT. We hypothesized that (1) compared to GH alone, treatment with GH plus PIO would result in better short-term glucose metabolism; and (2) compared to placebo, treatment with GH plus PIO would lead to greater reductions in both VAT and insulin resistance over time.

## METHODS

### Participants

A total of 81 overweight adult men and women were enrolled in the study. They were recruited between March 2003 and March 2004 using fliers posted at medical centers and other facilities throughout the San Francisco Bay Area. Specific criteria for inclusion were age between 40 and 75 years, BMI ≥ 27 kg/m^2^, and WC > 100 cm for men and > 88 cm for women. As part of the screening visit, a 75-g oral glucose tolerance test (OGTT) was performed following a ten- to 12-h overnight fast. Participants with IGT (fasting plasma glucose [FPG] <125 mg/dl and 2-h postprandial glucose = 140–200 mg/dl) were enrolled. Respondents were not eligible to participate if diabetes was known to exist or a screening OGTT revealed either a FPG ≥ 126 mg/dl or 2-h postprandial glucose (PPG) ≥ 200 mg/dl that was repeated and confirmed on separate day. Other exclusion criteria included the following: known history of malignancy or congestive heart failure; recent treatment with weight-reducing medications or corticosteroids in doses exceeding standard replacement; or being of child-bearing potential and either breastfeeding or declining contraception throughout the treatment period. In addition, respondents with a history of acromegaly or clinically significant cardiac, pulmonary, hepatic, or renal disease, serum alanine aminotransferase (ALT) more than three times above the upper normal limit, or uncontrolled hypertension were excluded.

### Study Design

The total treatment period for this randomized, double-blind, double-dummy, parallel group study with a 2 × 2 factorial design was 40 wk. A combination of two drugs was used: PIO (Actos 30 mg/d, Takeda Pharmaceuticals, http://www.tpna.com) or its placebo and recombinant human GH (Nutropin AQ 8 μg/kg/d, Genentech, http://www.gene.com) or its placebo. Enrolled participants were randomly assigned by a third-party investigator to receive one of the following treatment combinations: GH + PIO, GH + PIO placebo, GH placebo + PIO, or GH placebo + PIO placebo (Table 1). All testing and follow-up were conducted at the Clinical Studies Unit of the Veterans Affairs Palo Alto Health Care System, Palo Alto, California. Ethical approval for this study was provided by the Administrative Panel on Human Subjects in Medical Research at Stanford University, and all participants provided written informed consent.

**Table 1:**
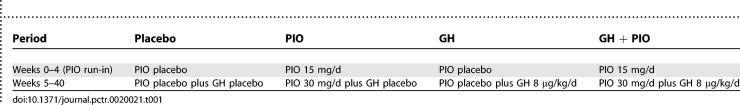
Treatment Assignment of Participants

### Randomization: Sequence Generation

Generation of the randomization sequence was performed at the office of a third-party investigator not affiliated with the study. Permuted blocks and stratification by BMI, gender, 2-h postprandial glucose (after 75-g oral glucose challenge), and estrogen repletion status of women were used.

### Randomization: Allocation Concealment

The randomization list generated in the office of the third-party investigator was forwarded to an investigational pharmacist, who dispensed medications. All randomization assignments were sequentially numbered and placed in sealed opaque envelopes. Assignment codes were fully concealed until after all recruitment, testing, and data analyses were complete.

### Randomization: Implementation

After being evaluated for eligibility at screening, participants who met criteria for inclusion into the study were assigned a code comprising a number and three letters. The number corresponded to the order of each participant's enrollment into the study. This code was forwarded along with relevant information for stratification to the third-party investigator. The structure of the sequence of randomization was unknown to the researchers involved with the study. Randomization assignment for each participant was forwarded only to the investigational pharmacist, who dispensed all medications.

### Masking

Given the double-blind, placebo-controlled nature of the study, all participants and investigators were blinded to group assignment until after the study was completed and data were analyzed. The manufacturers of recombinant human GH and PIO provided matching placebos that were indistinguishable from the active medications. Medication seals, labels, and containers were utilized in a uniform fashion to preserve the study blind.

### Sample Size

Sample size was determined using data from Johannsson et al. [19], in which nine months of GH resulted in a 17.9% ± 3.5% reduction in visceral fat along with an improvement in insulin sensitivity in abdominally obese men. Using this information, we determined that 12 participants were needed per group to have an 80% chance of detecting a change in visceral fat of at least 17.9%. However, since women were also included in the study and are known to respond less effectively to GH than men, we increased the sample size to 15.

### Outcomes

The primary outcome measures were change in visceral fat content and change in insulin sensitivity. At baseline (wk 0) and after 40 wk of total treatment, computed tomography (CT)-scan measurements were performed to quantify visceral fat area. Baseline versus post-treatment insulin sensitivity was assessed using a 3-h insulin suppression test.

Secondary outcome measures included assessments of short- and long-term glucose metabolism, BMI, and anthropometric measurements. FPG was measured monthly, and baseline versus post-treatment glucose tolerance was measured using a 3-h OGTT. Glycohemoglobin was measured at baseline and every three months thereafter at the clinical laboratory of the Veterans Affairs Palo Alto Health Care System. Body weight was measured monthly, and measurements of WC and waist-to-hip ratio (WHR) were performed at baseline and again at week 40.

Post-treatment OGTT and insulin suppression testing were performed on separate days following a 3- to 4-wk washout of study drugs. A drug washout was used to facilitate a direct assessment of the relationship between change in body composition and change in glucose disposal over time without the potential confound of direct drug effects on these measurements.

### Ancillary Tests

Lipids were measured using available stored serum samples from baseline and post-treatment visits. This was the only analysis performed that was not prespecified in the protocol. Samples from participants who began treatment with lipid-lowering medications anytime during or shortly before their participation in the study were not assessed for lipid measurements. Triglycerides, HDL cholesterol, and total cholesterol were measured directly, and LDL cholesterol was calculated based on the method described by Friedewald et al. [26]. Measurements were performed in the clinical laboratory of the Veterans Affairs Palo Alto Health Care System.

### Safety Measures

Participants were followed monthly as outpatients and had a complete physical examination at each visit. Each participant was examined for treatment-related side effects, including peripheral edema or other fluid retention symptoms. Serum insulin-like growth factor 1 (IGF-1) levels were obtained at baseline and every two months after starting injections until the end of the treatment period (week 40). An immunochemiluminometric assay in a reference laboratory (LabCorp, http://www.labcorp.com) was used to measure serum IGF-1. ALT levels were obtained at baseline and every 2 mo thereafter.

Dose adjustments for either GH or PIO were made if any of the following occurred during treatment: FPG > 140 mg/dl without associated symptoms, serum IGF 1 exceeding the upper normal reference limit for young adults, or fluid retention symptoms such as arthralgias, or carpal tunnel syndrome. Participants were discontinued if any of the following occurred during treatment: symptoms associated with FPG increase, an increase in serum ALT to more than three times the upper reference limit, congestive heart failure, or persistence of side effects despite GH or PIO dose reduction.

### Diet, Exercise, and Activity

Participants were asked to adhere to their usual daily dietary consumption and exercise routine for the duration of the study. They were instructed to report any significant changes in dietary intake or physical activity.

### Anthropometric Measurements

Weight was measured to the nearest 0.1 kg on a standard scale, and height was measured in centimeters using a wall-mounted stadiometer. BMI was calculated as the body weight (kg) divided by the height (m^2^). A standard tape measure was used to quantify waist and hip circumferences as well as the WHR ratio. WC was obtained while the participant was supine and with the tape measure placed at the level of the umbilicus. Hip circumference was obtained while the participant was standing and with the tape measure placed at the level of greatest gluteal protrusion observed from the side of the participant.

### CT Scan of the Abdomen

A noncontrast CT scan was performed to quantify abdominal subcutaneous and visceral fat area. This imaging technique is among the most reliable for measuring visceral fat [27]. With the individual in a supine position, a preliminary scout film of the abdomen and pelvis was obtained to identify anatomic landmarks. A 0.8-mm thick CT slice was obtained at the level of the L4–5 lumbar disc, as visceral fat area at L4–5 has been shown to correlate with total visceral fat volume. Fat content was represented in Hounsfield units in the range of −150 to −50, and visceral fat and subcutaneous fat regions were identified as described previously [28]. All fat measurements were performed using Image J Software, National Institutes of Health, (http://rsb.info.nih.gov/ij) and are recorded in centimeters squared. A single General Electric LightSpeed Scanner (http://www.ge.com) at the Veterans Affairs Palo Alto Health Care System was used to perform all baseline and week-40 CT scans.

### OGTT

After an overnight fast lasting ten to 12 hours, participants arrived at the Clinical Studies Unit and had an FPG measurement. Participants then ingested a 75-g oral glucose beverage and had additional plasma glucose measurements 30 min, 60 min, 120 min, and 180 min after beverage ingestion. All glucose measurements were performed using an on-site glucose analyzer (Analox Instruments, http://www.analox.com). Specimens were centrifuged immediately, and the on-site analyzer was calibrated daily. The five OGTT glucose measurements were used to calculate the glucose area under the curve (AUC) by the trapezoidal method.

### Insulin Suppression Test

Insulin sensitivity was measured using a three-hour insulin suppression test [29–31]. After fasting for ten to 12 h, participants arrived at the Clinical Studies Unit and an intravenous catheter was placed into each antecubital vein. One catheter was used to draw blood for glucose measurements, and the catheter in the contralateral arm was used for infusion of octreotide, insulin, and 20% dextrose solutions. The three infused substances were administered simultaneously. Octreotide acetate (Sandostatin, Novartis Pharmaceuticals, http://www.novartis.com) was used to suppress endogenous secretion of insulin and was infused at 0.27 μg/m^2^/min. Insulin was infused at 32 mU/m^2^/min, and 20% dextrose was infused at 267 mg/m^2^/min. Plasma glucose was measured every 30 minutes for the first 150 min and then every 10 min during the last half hour of the test. The last four serum glucose measurements were then averaged and constituted the individual's SSPG. The SSPG provides a direct measure of insulin-mediated glucose uptake and can be used to describe each individual's insulin sensitivity [29–31].

### Statistical Methods

Using all available data from all participants who had a follow-up measurement of visceral fat area and insulin sensitivity, regardless of adherence to treatment, a modified intention-to-treat analysis was used to evaluate data. The last available postrandomization value obtained prior to a missing measurement was carried forward for the missing data. Data were analyzed using GB-Stat statistical software for Macintosh (Dynamic Microsystems, http://www.gbstat.com/macintosh/index1.htm). Data for primary and secondary analyses are expressed as the mean ± standard error of the mean (SEM). One-way ANOVA was used to compare baseline values of the participant groups. For measurements pertaining to visceral fat, anthropometrics, insulin sensitivity, glucose and lipoprotein metabolism, ANOVAs for repeated measures were used to analyze differences between data obtained at all time points. Pearson's correlation coefficient was used to determine the relationship between change in VAT and change in insulin sensitivity. Results are considered statistically significant at *p* < 0.05.

## RESULTS

### Participant Flow

**Figure 1:**
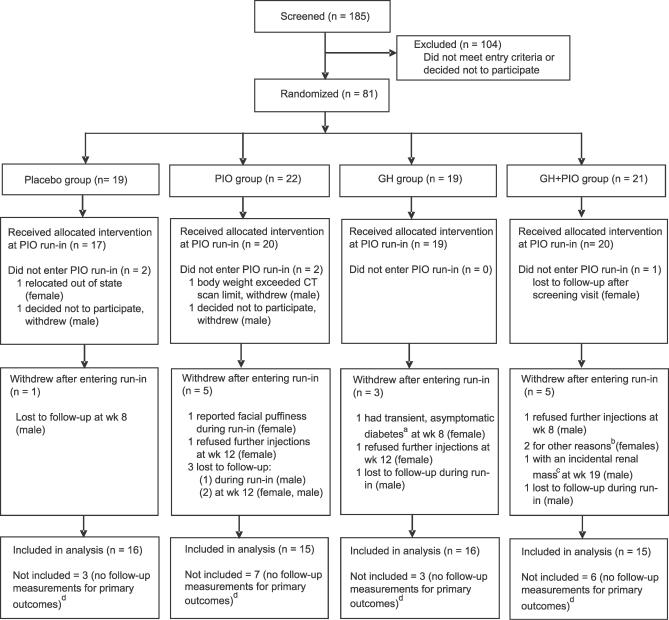
Screening, Randomization, and Follow-Up ^a^FPG reverted to pretreatment levels within 1 mo after stopping GH. ^b^Indicated at week 12 that they could no longer adhere to follow-up schedule for personal reasons. ^c^Reported as a renal cell carcinoma after removal. In a CT scan done before GH was initiated, the solitary mass appeared completely unchanged and was indistinguishable from colon. ^d^Did not have a follow-up CT scan and 3-h insulin sensitivity test after an overnight fast.

We randomized 81 participants to one of four treatment groups (Figure 1). There were 19 African Americans, five Hispanics, two Pacific Islanders, two Asians, and 53 individuals of European descent. A total of 76 participants received allocated treatment after entering the PIO run-in period. At baseline, no significant differences in age, BMI, gender, estrogen status of female participants, or glucose levels were present between the groups (Table 2). A total of 19 withdrew or were lost to follow-up, and postrandomization measures of visceral fat and insulin sensitivity after a confirmed overnight fast were not obtained. We gave follow-up tests of visceral adiposity and insulin sensitivity to 62 participants, and these were included in the final data analyses. Group assignment, follow-up, and withdrawals are shown in Figure 1.

**Table 2:**
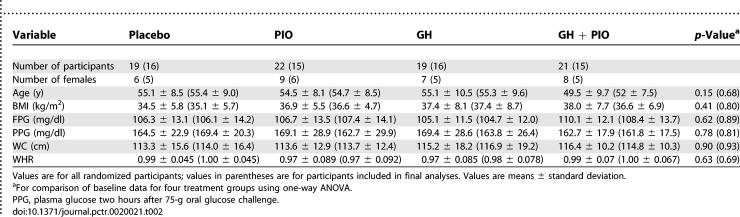
Characteristics of Participants at Baseline

### Outcomes and Estimation

#### Primary outcome measures: VAT.

**Figure 2:**
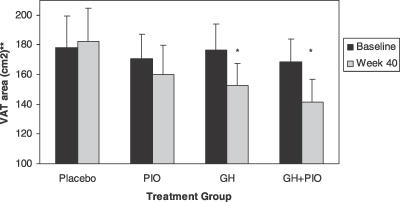
Change in VAT Area after 40 Weeks of Treatment with GH and/or PIO in Abdominally Obese Adults with IGT *p = 0.01 for within-group comparison of VAT area at week 40 versus baseline. **p = 0.03 for comparison of VAT areas for four treatment groups combined using analysis of variance for repeated measures. Values are means ± SEM.

As shown in Figure 2, 40 wk of GH treatment resulted in significant reductions in visceral fat area. After 40 wk of treatment, visceral fat area declined 23.9 ± 7.4 cm^2^, a mean difference of −28.1 cm^2^ compared with placebo (95% confidence interval [CI] −49.9 cm^2^ to −6.3 cm^2^; *p* = 0.02) (Table 3). A similar decline in VAT was seen in the GH + PIO group versus placebo, with a mean difference of −31.4 cm^2^ (95% CI −56.5 cm^2^ to −6.3 cm^2^; *p* = 0.02). The corresponding percentage declines in visceral fat in the GH and GH + PIO groups were 13.1% and 16.6%, respectively, indicating the lipolytic effect of GH on this adipose tissue depot. In contrast, VAT area did not decline significantly in the PIO group (6.4%).

**Table 3:**
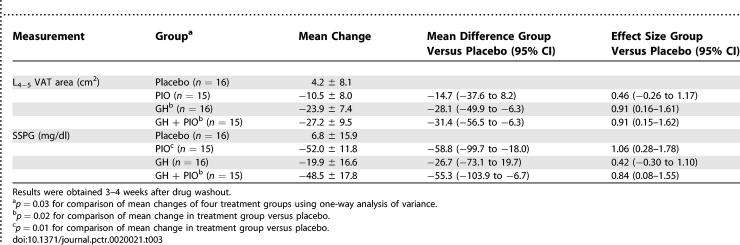
Mean Change (± SEM) in VAT and Insulin Sensitivity by Treatment Group

#### Primary outcome measures: Insulin sensitivity.

**Figure 3:**
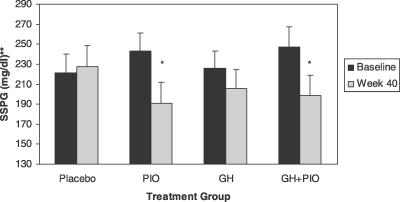
Change in SSPG after 40 Weeks of Treatment with GH and/or PIO in Abdominally Obese Adults with IGT Values are means ± SEM. *p = 0.001 and 0.01, respectively, for PIO group and GH + PIO group for within-group comparison of week 40 versus baseline SSPG. **p = 0.03 for comparison of SSPG for four treatment groups combined using analysis of variance for repeated measures.

Treatment with PIO improved endogenous insulin sensitivity in abdominally obese, insulin-resistant individuals with IGT, as seen in Figure 3. Insulin resistance, assessed by SSPG, declined 52 ± 11.8 mg/dl with PIO, a mean difference of −58.8 mg/dl compared with placebo (95% CI −99.7 mg/dl to −18.0 mg/dl; *p* = 0.01) (Table 3). Similarly, SSPG declined when both GH and PIO were combined, with a mean difference compared with placebo of −55.3 mg/dl (95% CI −103.9 to −6.7 mg/dl; *p* = 0.02). The corresponding percentage reductions for SSPG in the PIO and GH + PIO groups were similar: 21.4% in the PIO group and 19.6% in the GH + PIO group. This indicates an important role for PIO in ameliorating insulin resistance. Despite a reduction in visceral fat content, SSPG did not decline significantly following prolonged GH treatment (8.8%).

#### Secondary outcome measures and ancillary tests: Serum IGF-1 levels.

**Figure 4:**
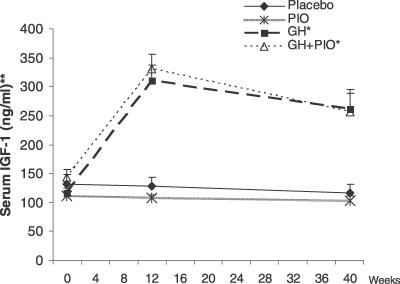
Effects of Four Different Interventions on Serum IGF-1 in Abdominally Obese Adults with IGT Values are means ± SEM. *p < 0.01 for within-group comparison of IGF-1 at follow-up versus baseline time points. **p < 0.001 for comparison of IGF-1 values for four treatment groups combined using analysis of variance for repeated measures.

At baseline, serum IGF-1 levels were similar between treatment groups (Figure 4). After nine months of GH treatment, serum IGF-1 in the GH group increased to 262.2 ± 35.1 ng/ml (an increase of 145.9 ± 33.3 ng/ml above baseline IGF-1) and to 256 ± 36.2 ng/ml in the GH + PIO group (an increase of 112.1 ± 34.8 ng/ml above baseline IGF-1). In both GH-treated groups, the IGF-1 increases generally did not exceed 2 standard deviations above the age-adjusted reference mean (unpublished data). No within-group changes in serum IGF-1 occurred in the placebo or PIO groups over time.

### BMI, Anthropometrics, and Other CT Measurements

The effects of treatments on body composition are summarized in Table 4. No significant changes in subcutaneous fat area were seen among the treatment groups. In addition, BMI, WC, and WHR did not change significantly in any of the treatment groups, except for a 7.0% decline in WHR that occurred in the GH + PIO group.

**Table 4:**
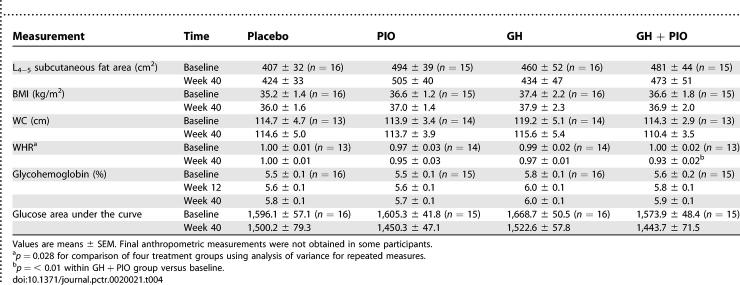
Secondary Outcome Measures Related to Body Composition and Glucose Metabolism

### Glucose Metabolism and Lipids

**Figure 5:**
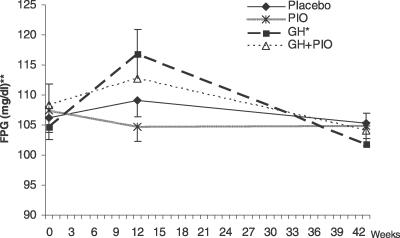
Effects of Four Different Interventions on Fasting Blood Glucose in Abdominally Obese Adults with IGT Week 43 FPG was obtained three to four weeks after drug washout. Values are means ± SEM. *p = 0.01 for within GH group comparison of week 12 versus baseline FPG. **p = 0.048 for comparison of four treatment groups combined before and after 12 weeks of treatment using analysis of variance for repeated measures.

The effects of treatments on short- and long-term glucose concentrations are summarized in Table 4. In the GH group only, FPG increased from 104.7 ± 2.8 mg/dl to 116.8 ± 3.9 mg/dl at week 12 (*p* < 0.01) (Figure 5). However, mean glycohemoglobin levels for all groups did not change significantly during follow-up. In addition, baseline versus post-treatment mean glucose area under the curve were similar for all treatment groups. Based on the final OGTT (performed after a 3- to 4-wk drug washout for both GH and PIO), two participants in the placebo group and two participants in the PIO groups had newly diagnosed diabetes. No new cases of diabetes were identified in either GH-treated group at the end of the study.

Although lipid testing was not prespecified in the protocol, measurement of lipids was performed using available stored serum samples. No significant differences in baseline versus post-treatment triglycerides, HDL cholesterol, LDL cholesterol, or total cholesterol were seen between the four treatment groups (Table 5).

**Table 5:**
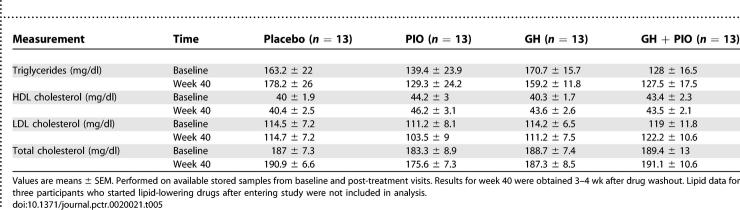
Lipid Measurements

### Correlations of SSPG Changes with Body Composition Findings

When participants from all four treatment groups were combined, change in SSPG was most directly related to change in VAT (*r* = 0.425, *p* < 0.001). There were no other significant correlations between SSPG and subcutaneous fat, BMI, anthropometric measurements, or measurements of glucose and lipoprotein metabolism.

### Adverse Events

Side effects observed in the trial are fully summarized in this section and in Table 6. Side effects were generally mild and related to fluid retention. Fluid retention symptoms resolved in all cases either spontaneously or following a dose reduction in GH or PIO. In addition, five participants in the GH group experienced transient increases in fasting glucose concentrations about 4–8 wk after starting injections. The glucose increases were not associated with symptoms and resolved either spontaneously (two participants), after a 25% reduction in GH dose (two participants), or after GH was briefly held for 3–4 wk (one participant). Except in the latter case, glucose increases above baseline were modest and did not exceed 5–15 mg/dl. No new cases of diabetes were identified in either GH-treated group after 40 wk of treatment. During follow-up, no significant change in ALT was seen in any group (unpublished data).

**Table 6:**
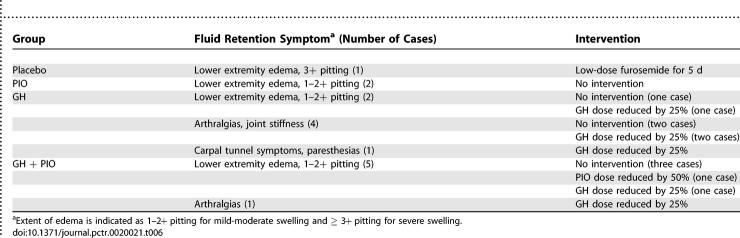
Fluid Retention Symptoms and Interventions

## DISCUSSION

### Interpretation

In the present study, nine months of treatment with GH resulted in a reduction in VAT. The well-established lipolytic effect of GH has been attributed to suppression of lipoprotein lipase activity or enhanced responsiveness of adipocytes to catecholamine [20,32–34]. Fasting glucose increased during the first four to eight weeks of GH treatment, but this resolved either spontaneously or shortly after GH dose reduction. No significant differences between baseline and post-treatment FPG and OGTT-derived glucose area under the curve were seen following prolonged GH administration. Lipoprotein levels did not change despite a significant reduction in visceral fat content. Although lipid measurements were performed after a three-week washout of study drug, possibility altering the benefit on lipids, trials assessing the effects of prolonged GH administration on lipids have yielded conflicting results [19,20, 35–37].

Insulin sensitivity did not improve after nine months of GH despite VAT reduction. Although GH is known to impair insulin action directly by reducing phosphorylation of insulin receptor substrate-1 (IRS-1) and IRS-2 proteins [38,39], a long drug washout was used to reduce possible confounding effects of GH and IGF-1 on glucose disposal [40]. A possible explanation for the lack of SSPG change is that visceral fat declined 13.1% in the GH group, less than the 18% reduction in visceral fat that was associated with an improvement in insulin sensitivity in Johannsson et al. [19]. Unlike the previous investigation, our sample included five women (approximately one-third of the total sample), and GH-treated women are known to achieve smaller reductions in visceral and subcutaneous fat than men [41–43]. The diminished effect of GH on body composition in women has been attributed to the estrogen-induced antagonism of GH action [41–43]. In addition, although mean BMI did not change and participants were asked to adhere to their usual diet and exercise routine, dietary and lifestyle factors are a potential confound. The lack of rigorous monitoring precluded the maintenance of a stable weight in all participants, and weight gain did occur in individual cases. It has been reported that the lipolytic effect of GH is blunted in individuals who experience weight gain as a result of increased dietary intake or decreased exercise, or both [20].

We found that 40 weeks of PIO improved insulin sensitivity in individuals with IGT. As with patients with type 2 diabetes [44], the insulin-sensitizing effect of PIO persisted several weeks after the drug was stopped in PIO-treated individuals. However, no significant change in body composition, including visceral or subcutaneous fat content, was seen.

In vitro and in vivo studies have indicated that activation of peroxisome proliferator-activated receptors gamma with TZDs improves insulin sensitivity by modulating the expression of various genes in the insulin-signaling pathway and increasing the production of glucose transporter proteins [45–47]. Miyazaki et al. recently suggested that PIO's beneficial effect on insulin action in type 2 diabetes might be related to a change in body composition [23]. The authors indicated that a shift in body fat from visceral to subcutaneous fat depots was observed following 16 weeks of PIO, and the change was associated with an improvement in hepatic and peripheral insulin sensitivity. The greater reduction in visceral fat reported in Miyazaki et al. [23] may have been related to the makeup of the study population (patients with type 2 diabetes versus those with IGT) or the use of a higher PIO dose (45 mg/d instead of 30 mg/d). However, other investigators reported no change in visceral fat content in PIO-treated diabetics [48]. At present, the overall effect of TZDs on visceral fat content remains uncertain.

Our sample size was likely not large enough or adequately powered to detect significant changes in glucose concentrations during PIO treatment [49]. Similarly, with regard to multiple testing, the analysis of lipids was performed as an ancillary test that was not prespecified in the protocol. The lack of significance in lipid changes with PIO in the present study was seen in the context of a smaller subset of data analyzed (only 13 participants per group). Therefore, the possibility that the study was not adequately powered to see changes in lipid parameters with PIO must be considered, given that improvements in triglyceride and HDL cholesterol have been reported in PIO-treated diabetics [50–51]. It is unclear, however, if PIO exerts different effects on lipids in diabetic versus nondiabetic adults such as the participants in this study.

In the GH plus PIO group, mean FPG remained stable, and both visceral fat and insulin resistance declined over time. PIO attenuated the diabetogenic effect of GH shortly after GH injections began and before any change in body composition would have been expected. Further, the reduction in visceral fat area that occurred after 40 weeks of GH plus PIO treatment (27 ± 4 cm^2^) was similar to the reduction that occurred with GH alone (24 ± 7 cm^2^). Only cotreatment with PIO resulted in improved insulin sensitivity, while GH alone did not. These findings suggest an important role for TZDs in improving insulin sensitivity [44].

### Overall Evidence

The findings of this study can be reviewed in the context of other similar clinical trials. We performed a Medline search for clinical trials involving treatment with GH, a TZD, or both in overweight and/or insulin-resistant adults who were nondiabetic and were otherwise healthy. To facilitate better comparisons with the current study, the search was restricted to trials in which abdominal and visceral fat was directly measured using CT scan or MRI, and the pharmacologic intervention (GH or TZD) was not combined with lifestyle modification. Randomized controlled trials performed in a community setting were identified. Our search (up to 1 February 2007) revealed eight publications that involved four GH-treated cohorts and three TZD-treated cohorts.

In the GH trials [19,20,43,52,53], controls received a placebo and treatment duration ranged from five weeks to one year. Total abdominal or visceral fat declined significantly in men [19,43], while in women, a decline versus placebo was seen in some [20,52], but not all [46] studies. In addition, the magnitude of fat reduction observed with GH was consistently greater in men than in women. Differences in glucose metabolism were seen among studies that reported these measured data. Except in one study [20], short-term administration of GH transiently worsened insulin resistance [19,53] and increased fasting glucose levels [53]. However, prolonged GH administration was not associated with deleterious changes in glucose tolerance or insulin sensitivity [19,20], as was the case in the present study. The results for lipid data are conflicting, with total cholesterol after six months of GH reported to be unchanged versus baseline in men [19] and transiently decreased and subsequently returning to baseline after one year of treatment in women [20].

In the TZD trials [54–56], treatment duration ranged from ten to 20 weeks. We found two publications that involved PIO [54,55], and one that involved troglitazone [56]. The control groups received either a placebo [56], metformin [55], or diet and exercise [54]. Insulin sensitivity improved in all cases [54–56], indicating a potentially beneficial role for TZDs in overweight, nondiabetic, healthy adults. Conversely, visceral fat content did not decline significantly versus baseline or controls in any of the studies. In the study that compared TZD treatment to placebo [56], no significant changes in fasting or postprandial glucose levels were reported. Although triglycerides decreased and LDL increased in troglitazone-treated participants [56], changes in lipoprotein levels over time were not seen with PIO in healthy, overweight, nondiabetic volunteers [54,55].

In the current study, combined treatment with GH and PIO resulted in estimated effect sizes for ΔVAT and ΔSSPG that were similar to GH and PIO, respectively. We considered whether the effect sizes seen in this trial might be an artifact of multiple testing for statistical significance, but this was not thought to be important because (1) sample size and power calculations were determined to see true differences in primary outcome measures; and (2) the reduction in visceral fat seen with GH (13.1%) and the decline in insulin resistance with PIO (21.4%) are comparable with the results of others and have been consistently well-documented in various studies [19,20, 52–56]. Taken together, these findings suggest that PIO improves insulin sensitivity in IGT adults and complements the lipolytic effect of GH.

### Generalizability

Moderate weight loss (2.3–4.5 kg) induced by diet and/or exercise has been associated with reductions in insulin resistance and VAT of as much as 15%–20% or more, similar to that seen with GH + PIO in the present study [9]. VAT reduction and improved insulin action may not only be important for preventing or delaying diabetes, but also for modulating surrogate markers for cardiovascular disease and improving cardiovascular outcome [8–10].

Given the rising costs and growing burden of diabetes on the health-care system [57], strategies that aim to prevent or delay the onset of this disease might be beneficial [58]. Although diet plus exercise is frequently used in experimental settings and is the mainstay for body fat reduction, this approach often fails in clinical situations and is commonly associated with recidivism. For pharmacologic interventions such as GH that might be useful in reducing body fat, cost and side effects are important considerations. The average cost for GH replacement in adults is widely regarded as unacceptably high. However, the cost has declined in recent years, in part because of evolving dosing strategies that have steadily lowered initial GH doses used to achieve similar efficacy [59]. Similarly, GH-associated side effects are generally transient or are well tolerated, and treatment discontinuation is not common, particularly with the lower GH doses that have been advocated in recent years [59]. Further, the issue of cost with the use of GH in obesity must be considered in the overall context of the soaring costs and rising health-care burden of diabetes. Given the link between visceral obesity, insulin resistance, and the risk for diabetes and cardiovascular disease, future studies assessing the body composition and metabolic effects of GH + PIO in adults with the metabolic syndrome may be warranted.

## SUPPORTING INFORMATION

CONSORT Checklist (49 KB DOC)

Trial Protocol (287 KB DOC)

## Abbreviations

ALT: alanine aminotransferase
BMI: body mass index
CI: confidence interval
CT: computed tomography
FPG: fasting plasma glucose
GH: growth hormone
IGF-1: insulin-like growth factor 1
IGT: impaired glucose tolerance
OGTT: oral glucose tolerance test
PIO: pioglitazone
SEM: standard error of the mean
SSPG: steady-state plasma glucose
TZD: thiazolidinedione
VAT: visceral adipose tissue
WC: waist circumference
WHR: waist-to-hip ratio
