# Supplementary material for: Effects of Growth Hormone and Pioglitazone in Viscerally Obese Adults with Impaired Glucose Tolerance: A Factorial Clinical Trial
Source: PLoS Clin Trials. 2007 May 4;2(5):e21. doi: 10.1371/journal.pctr.0020021 (PMC1865086; doi:10.1371/journal.pctr.0020021)
Supplement: Trial Protocol — (287 KB DOC) [file pctr.0020021.sd002.doc]

**SUPPORTING INFORMATION: Trial Protocol**

# Effects of rhGH and Pioglitazone in Viscerally Obese Adults with IGT

A. **ABSTRACT**

People with impaired glucose tolerance (IGT) who lose visceral fat also tend to lower their risk of developing diabetes. This benefit occurs because visceral fat reduction improves insulin sensitivity and reduces postprandial glucose levels. Thiazolidinediones (TZDs) and growth hormone (rhGH) taken independently for an *extended period of time* can reduce visceral fat and improve endogenous insulin sensitivity. However, in the *short-term* (ie: first 6 weeks of treatment) rhGH may increase insulin resistance. In contrast, a TZD added to rhGH prevents short-term rhGH-induced insulin resistance in experimental animals, suggesting a possible therapeutic benefit with this drug combination in humans. In addition, like rhGH, TZDs mediate some of their insulin sensitizing effects by reducing visceral fat depots. It is therefore possible that the addition of a TZD with standard growth hormone therapy will optimize the short-term and long-term improvements in body composition, insulin sensitivity and glucose metabolism in humans.

The specific aims of this study are as follows: (1) to assess the effects of TZDs with and without rhGH on visceral fat content, insulin sensitivity and postprandial glucose levels in viscerally obese subjects with IGT; *and* (2) to compare the effects of TZDs *plus* rhGH with rhGH alone on short-term glucose metabolism. Sixty adult men and women between the ages of 40 and 75 will be recruited to participate and will receive a TZD and rhGH in a double-blind manner. During weeks 0-4, a “run-in” period, subjects will receive a TZD or oral placebo. During weeks 5-40, a TZD or oral placebo will be co-administered with rhGH or a subcutaneous placebo in a 2 X 2 study design. Short-term glucose metabolism will be monitored using fasting glucose levels. Changes in visceral fat content will be quantified using abdominal CT scanning and anthropometric measurements. At baseline and after 9 months of combination drug or placebo treatment, serum postprandial glucose levels will be measured using a standard oral glucose tolerance test, and endogenous insulin sensitivity will be measured using a hyperinsulinemic euglycemic clamp.

It is expected that the combined use of a TZD and rhGH will be more efficacious in reducing visceral fat quantity and improving insulin sensitivity in viscerally obese adults with IGT compared with the use of either drug alone. The potential long-term benefit of this finding is a reduction in the incidence of type 2 diabetes in populations at high risk of developing diabetes, such as individuals with visceral obesity and IGT.

B. **HYPOTHESES AND SPECIFIC AIMS TO TEST HYPOTHESES**

***Hypothesis #1:*** Subjects who receive rhGH and pioglitazone for 9 months will achieve greater visceral fat reduction, improved insulin sensitivity and lower postprandial glucose levels compared with subjects who receive placebo.

***Specific aim to test hypothesis #1:*** We will assess the effects pioglitazone with and without prolonged rhGH administration on visceral fat change and determine whether change in visceral fat quantity is associated with changes in endogenous insulin sensitivity and postprandial serum glucose levels.

***Hypothesis #2:*** Subjects who receive pioglitazone *and* rhGH will demonstrate less transient worsening in glycemic control during the first 6 weeks of treatment compared with subjects who receive rhGH alone.

***Specific aim to test hypothesis #2:*** We will compare the effects of a TZD *plus* rhGH with rhGH alone on short-term glucose metabolism. In doing so, we will also determine whether the co-administration of a TZD can effectively counteract the expected transient rise in insulin resistance associated with rhGH and help to maintain better short-term postprandial glucose control compared with rhGH alone.

C. **BACKGROUND**

***Link between visceral fat and cardiovascular disease:***   In recent years, we have gained a better understanding of the relationship between cardiovascular (CV) disease and various components of body fat such as total body, subcutaneous and intra-abdominal (or visceral) fat. Several epidemiological studies have indicated that, of these components, visceral fat – a combination of omental and mesenteric adipose tissue – is the greatest predictor of CV disease and events. Rexrode et. al prospectively followed 45,000 older women over 8 years to determine the effect of visceral fat on CV outcome. Women with android obesity – that is, a large waist circumference (ie: >98.5 cm) and a large waist-to-hip ratio (ie: > 0.88) – had a more than twofold increased risk of coronary heart disease even after adjustment for hypertension, diabetes and high cholesterol levels, and even when body mass index (BMI) was normal (1).

Conversely, several prospective studies have indicated that a selective reduction in visceral fat reduces CV disease risk by improving hypertension, dyslipidemia, diabetes and other components of intermediary metabolism (2-7).

***Link between visceral fat and impaired glucose tolerance:*** Normal glucose homeostasis is maintained by a combination of sufficient insulin release from pancreatic islet cells and adequate tissue sensitivity to insulin. Insulin sensitivity is often diminished with visceral obesity, and when the pancreas fails to release enough insulin to overcome this resistance to insulin, a condition known as impaired glucose tolerance, or IGT, develops.

IGT is a pre-diabetic condition that is very common in viscerally obese adults and is defined by abnormally high postprandial glucose levels after a 75-gm oral glucose load (8). It is associated with adverse health consequences, including an increased risk of developing type 2 diabetes and an increased risk of cardiovascular disease (9-11). The exact cause of the increased CV risk in adults with IGT is unknown, but the postprandial hyperglycemia itself may be responsible.  Postprandial hyperglycemia appears to generate oxidative stress, increase protein glycation, increase hypercoagulability, and damage endothelial cells ­- all of which lead to atherosclerosis (12).

Visceral fat loss reduces postprandial glucose levels in IGT patients (13-19). Fujioka et. al showed that weight loss - and visceral fat reduction in particular - results in a significant reduction in postprandial glucose levels in viscerally obese women with IGT (20). Eriksson et. al also prospectively showed that IGT subjects achieved lower postprandial glucose levels after weight loss, and that this reduced the overall risk of developing type 2 diabetes (21). In addition, recently published data from the Diabetes Prevention Program (DPP) study indicate that a 5-7 % weight loss in IGT subjects reduces the risk of developing type 2 diabetes by 58% (22).

***GH, visceral fat reduction and enhanced insulin sensitivity:*** Growth hormone (GH), a glycopeptide hormone produced in the anterior pituitary, helps to maintain normal body composition and reduce visceral fat in adults (23). It also appears to have various effects on glucose, lipid and bone metabolism.

Viscerally obese adults have below normal integrated GH concentrations as measured by an ultrasensitive chemiluminescence assay (24). The association between visceral obesity and an apparent “underproduction” of GH has recently prompted several investigators to study the effects of rhGH on body composition and metabolism in viscerally obese adults. In a recent study, Johannsson et. al investigated the effects of rhGH in insulin resistant, viscerally obese men with normal glucose tolerance (25). As expected, subjects receiving rhGH lost a substantial amount of visceral fat – almost 18%. In addition, while overall glucose levels did not change during the study, an interesting paradoxical effect on short-term versus long-term insulin sensitivity occurred in rhGH-treated subjects. RhGH-treated subjects developed a transient *decrease* in insulin sensitivity as expected during the first 6 weeks of therapy. However, after 9 months of rhGH treatment, the glucose disposal rate (GDR), a measure of tissue insulin sensitivity*, increased* by 1.2 +/- 0.7 mg/kg per minute compared to baseline (*P* < 0.05), indicating that insulin sensitivity increases – rather than decreases - with long-term rhGH administration.

    How does GH cause a *transient* insulin resistance? GH exerts a direct antagonistic effect on the insulin signaling pathway in muscle cells. GH inhibits the insulin-induced activation of so-called insulin receptor substrate (IRS) proteins, and these “inactivated” proteins cannot trigger the usual cascade of events that ultimately lead to glucose uptake into tissues. Despite the antagonistic effects of GH on insulin, euglycemia is usually maintained because the pancreatic beta-cell increases insulin output in order to overcome tissue insulin resistance.

    What causes the subsequent increase in insulin sensitivity that occurs with prolonged rhGH administration? Visceral fat loss. This happens as follows: visceral fat, a vast depot of lipid-rich substances, releases large quantities of free fatty acids (or FFAs) into the circulation. FFAs cause insulin resistance by blocking the insulin-induced expression and cell surface translocation of proteins that mediate glucose transport into cells, called glucose transporter (or GLUT) proteins. A selective reduction in visceral fat, as occurs with prolonged rhGH administration, leads to diminished free fatty acid (FFA) flux, and this allows insulin-GLUT-mediated glucose disposal to occur. While rhGH directly antagonizes insulin in skeletal muscle, rhGH-induced visceral fat disappearance leads to enhanced insulin action in *multiple* tissue compartments, including adipocytes, skeletal tissue, hepatocytes, and other cells throughout the body. As a result, the indirect insulin “sensitizing” effects of visceral fat reduction with prolonged rhGH administration outweigh its direct insulin resistant effects, and the net result is a rise in endogenous insulin sensitivity (25).

    Visceral fat reduction and the insulin sensitive state that follows help to lower postprandial glucose levels in individuals with IGT. Postprandial glucose reduction occurs in this setting because insulin, now free from the antagonizing effects of FFAs, is able to turn off gluconeogenesis, or glucose production, in the liver and stimulate the uptake and clearance of glucose into muscle and other tissues. It is possible that similar improvements in glucose metabolism will occur in viscerally obese IGT patients who receive rhGH. However, this is theoretical. Prospective studies are needed to determine the effects of prolonged rhGH on glucose metabolism in this population.

***Thiazolidinediones plus rhGH: effects on glucose metabolism:*** Like rhGH, insulin sensitizer drugs known as thiazolidinediones help to improve endogenous insulin sensitivity – albeit by a somewhat different mechanism than rhGH. *Prolonged* rhGH improves insulin sensitivity primarily by reducing visceral fat content, which reduces free fatty acid flux in the circulation, thus removing a potent barrier to normal insulin signaling. TZDs are synthetic ligands that activate the nuclear receptor known as peroxisome proliferator activated receptor gamma, or PPAR gamma. The activation of PPAR gamma modulates the expression of various genes in the insulin signaling pathway. This, in turn, facilitates insulin’s action at the cellular level and allows glucose uptake to occur. The in vitro and in vivo insulin sensitizing effects of TZDs in adipocytes have been well characterized in animal models and are correlated with both PPAR gamma activation and increased expression of glucose transporter (or GLUT) proteins (26,27,28).

TZDs have been shown to completely overcome the *transient* insulin antagonizing effects of rhGH in animals (29). According to Sugimoto et. al 1998, rats treated with rhGH and a TZD maintained normal glucose homeostasis and insulin sensitivity during the period in which this drug combination was used, whereas rats treated with rhGH alone developed insulin resistance and abnormal glucose control (including impaired glucose tolerance). This study also demonstrated that the co-administration of a TZD (troglitazone) with rhGH helped to overcome the antagonizing effects of rhGH on insulin- mediated peripheral tissue glucose utilization and helped to restore insulin’s ability to suppress hepatic glucose production.

TZDs therefore may improve endogenous insulin sensitivity via several mechanisms, including PPAR gamma activation and increased expression of GLUT proteins in adipocytes and perhaps skeletal muscle and other tissues. In addition, a TZD called pioglitazone appears to improve insulin sensitivity by at least one additional very interesting mechanism: shifting or redistributing body fat from visceral fat depots to subcutaneous fat depots. Miyazaki and colleagues noted this finding in a recent study involving the treatment of insulin-resistant type 2 diabetics with pioglitazone (29a). In this study, 16 weeks of treatment with pioglitazone resulted in a reduction in visceral fat area of 9 % at L 4-5 compared to baseline (P <0.05) and a decline in the visceral fat: subcutaneous fat ratio of 25% at L 4-5 compared to baseline (P <0.01) (29a). The shift in fat content from the viscera to the periphery after 16 weeks of pioglitazone treatment was also associated with a statistically significant increase in hepatic insulin sensitivity in the postabsorptive state and higher total body glucose disposal rates as measured by the insulin clamp technique.

In summary, either rhGH or a TZD taken for a prolonged period can reduce visceral fat and improve endogenous insulin sensitivity. However, no human trials to date have prospectively assessed the combined effects of rhGH and a TZD over an extended period on visceral fat change, insulin sensitivity, and glucose homeostasis. It is anticipated that the combination of a TZD and rhGH will have an additive or synergistic effect in improving body composition and glucose homeostasis compared with either drug alone. This in turn would be very efficacious in delaying or preventing type 2 diabetes in viscerally obese adults IGT. It is also anticipated that TZDs will counteract the *transient* insulin antagonizing effects of rhGH and therefore allow this potent drug combination to be used to achieve its long-term benefits.

This protocol would be the first to test the hypothesis that viscerally obese IGT subjects who receive pioglitazone *and* rhGH will demonstrate at least 2 very important benefits: (a) in the short-term, less *transient* worsening in glycemic control compared with subjects who receive rhGH alone; and (b) in the long-term, a greater *reduction* in visceral fat quantity, insulin resistance and postprandial glucose levels compared with placebo or rhGH given alone.

D. **RESEARCH PLANS**

**Subjects:** Men and women between the ages of 40 and 75 with visceral obesity and IGT will be recruited to participate. This age group has a higher prevalence of both visceral obesity and IGT.

Specific inclusion criteria are as follows: (a) adults between the ages of 40 and 75; (b) fasting glucose less than 120 mg/dl PLUS 2 hour postprandial glucose level during 75 gram OGTT between 140 – 200 mg/dl; (c) BMI > 27; and (d) increased waist circumference and/or increased waist-to-hip ratio (*see anthropometric measurements* below).

Subjects will be free of major medical illnesses such as uncontrolled hypertension, congestive heart failure or active malignancies. Women of child-bearing potential who are or plan to become pregnant or will breastfeed an infant will be excluded. Subjects taking corticosteroids or other medications that can significantly alter glucose metabolism will also be excluded. Groups will be matched on gender, BMI, estrogen status, and 2-hr glucose level (during screening OGTT). A third party investigator will be responsible for all randomization and coding of medications and placebo.

#### SCREENING TESTS FOR SUBJECT SELECTION

**Physical exam:** Screening will include a complete medical history and physical examination. In addition, vital signs and blood pressure measurements will be performed.

**Anthropometric measurements:** Body mass index (in kg / height in meters squared), waist circumference (in inches), hip circumference (in inches), and waist-to-hip ratio (WHR) will be measured. The criteria for inclusion in this study is a BMI > 27 *plus* EITHER waist circumference > 40 inches in men or > 38 inches in women OR WHR >0.9 in men or >0.8 in women. These measurements are associated with both increased visceral adiposity and greater cardiovascular risk (2,30,31).

**Laboratory studies and ECG:** Liver function tests (LFTs) will be obtained in all subjects, and male subjects will have prostate specific antigen (or PSA) levels measured. Women of child-bearing potential will have a urine pregnancy test to exclude pregnancy. All blood and urine studies will be done at the VA Medical Center, Palo Alto, CA, and results will be compared to normal VA laboratory reference values. A supine electrocardiogram (ECG) will also be obtained.

**Oral Glucose Tolerance Test:** Once subjects have passed this background screening, they will be given an oral glucose tolerance test (OGTT). After an overnight fast, subjects will come to the VA and drink a 75 gm glucose beverage. Serum glucose levels will be measured at 0 min (fasting), 30 min, 60 min, 120 min and 180 min after glucose ingestion.

#### EXPERIMENTAL DESIGN

After screening, 60 men and women who meet the criteria for study inclusion will be randomly assigned into 4 groups, with each group containing 15 subjects (n=15). The treatment period will start at week 0 and continue for 40 weeks total.  **During** **weeks 0-4**, *groups 1 and 2* will receive an oral placebo and *groups 3 and 4* will receive pioglitazone orally every day. **During weeks 5-40**, *group 1* will continue the oral placebo and self-inject a SQ placebo and *group 2* will continue the oral placebo and self-inject rhGH. Also **during weeks 5-40**, *group 3* will continue pioglitazone and self-inject a SQ placebo and *group 4* will continue pioglitazone and self-inject rhGH.

*Group 1* (n=15): Oral placebo plus SQ placebo.

*Group 2* (n=15): Oral placebo plus rhGH.

*Group 3* (n=15): Pioglitazone plus SQ placebo.

*Group 4* (n=15): Pioglitazone plus rhGH.

Sample size was determined using data from Johannsson et. al, 1997 in which a dose of rhGH comparable to ours was used to achieve a 17.9 +/- 3.5 percent change in visceral fat volume and an increase in insulin sensitivity of 1.2 +/- 0.6 mg/dl • hr over time (24). Using this information, we have powered our study based on 2 primary efficacy endpoints as follows: (a) 12 subjects (n=12) are needed *per group* in order to have an 80% chance of detecting a change in visceral fat volume of at least 17.9 percent, and (b) 15 subjects (n=15) are needed *per group* in order to have an 80% chance of detecting a change in insulin sensitivity of at least 17.6 percent. Therefore, our sample size for this study will be 15.

**Study drugs:** The TZD that will be used is *pioglitazone hydrochloride*. Subjects in groups 3 and 4 will begin pioglitazone 15 mg orally every day for 8 weeks total. During weeks 9-12, subjects who have tolerated 15 mg pioglitazone daily will be increased to 30 mg/day and will remain at this dose for the duration of the study. However, the pioglitazone dose may be decreased or, in rare circumstances, increased if side effects such as peripheral edema or diabetes develop (please see *Pages 13 and 14* for TZD dose adjustments). Pioglitazone has been prospectively shown to reduce visceral fat content and improve insulin sensitivity in insulin resistant people. It is begun 4 weeks prior to rhGH in order to allow sufficient time for its insulin sensitizing effect to occur. RhGH-treated subjects will begin self-injecting rhGH 8 mcg/kg SQ every day at week 5 and will continue to do so for the duration of the study, unless side effects develop (please see *Pages 13 and 14* for rhGH dose adjustments). The total rhGH treatment period will be 9 months. Previous studies have indicated that this dosage and duration of treatment are generally well tolerated and effective in significantly reducing visceral fat.

**Diet, exercise and activity:** Subjects will adhere to their usual daily dietary consumption and exercise routine for the duration of the study. They will be asked to report any significant changes in dietary intake or physical activity.

**Physical exam:** A complete physical exam will be performed at baseline (week ­- 4 to 0) and again at weeks 4, 8, 12, 16, 20, 24, 32, 40 and 43. In addition, supine systolic and diastolic blood pressure will be measured in mmHg using a manual sphygmomanometer.

#### STUDY ENDPOINTS

**Oral glucose tolerance test:** As per the technique described under screening, a 75 gram OGTT will be repeated at week 43, approximately 3 weeks after discontinuation of all study medications (washout period). In addition, the glucose area under the curve, or AUC, will be measured using the trapezoid method. Based on previously published data, we expect that rhGH administration will cause at least a 10% reduction in both postprandial glucose (2 hours post OGTT) and glucose area-under-the-curve compared to baseline values.

**CT of the abdomen:** A non-contrast CT of the abdomen to quantify visceral adiposity will be obtained at the baseline visit and again at week 40. This imaging technique is among the most reliable for measuring visceral fat (32). Scans will be

obtained at 5 levels: spleen/liver, L3-4 lumbar disc, L4-5 lumbar disc, lower edge of pubic symphysis, and upper thigh (with reference to edge of lower symphysis). The area of the visceral and subcutaneous abdominal fat compartments at the

5 levels will be measured and recorded in centimeters squared. Abdominal visceral volume will then be calculated (in liters) using the visceral tissue areas of the 5 scans and the measured distances between them. The same CT scanner will be used at the start and completion of the study, and the same radiologist at VA Palo Alto will analyze all scans.

**Insulin Sensitivity Test (IST):** Endogenous insulin sensitivity will be measured using a 3-hr insulin sensitivity test (33,34). This test will be performed at baseline and again at week 43 (which follows a 3-week washout period for study medications). An intravenous catheter will be placed into each antecubital vein. One catheter will be used for blood draws for glucose measurements and the catheter in the contralateral arm will be used for infusion of octreotide, insulin and 20% dextrose. The 3 infused substances will be administered simultaneously. Octreotide acetate will be used to suppress endogenous secretion of insulin and will be infused at 0.27 µg/m^2^ per minute. Insulin will be infused at 32 mU/m^2^·min and glucose will be infused at 267 mg/m^2^ per minute. Serum glucose will be measured every 30 minutes for the first 150 minutes and then every 10 minutes during the last half hour of the test. The last 4 serum glucose measurements will averaged and recorded in mg/dl and will constitute the subject’s steady state plasma glucose (or SSPG). The SSPG provides a direct measure of insulin-mediated glucose uptake and will be used to describe each subject’s insulin sensitivity (33,34).

**Anthropometrics:** BMI, waist circumference and WHR will be measured at baseline and at weeks 8, 16, 32 and 40.

**IGF-1 levels:** Serum insulin like growth factor 1, or IGF 1, is a marker of GH status in the body. IGF 1 levels will be drawn at baseline and again at weeks 8, 16 and 32 and will be used along with clinical symptoms to assess rhGH dose adequacy. IGF 1 values greater than 2 standard deviations above the adult reference mean will result in a 50% rhGH dose reduction.

**Liver function tests, fasting glucose, hemoglobin (Hgb) A1C:** LFTs will be obtained at baseline and again at weeks 8, 16, 24, 32 and 40. Fasting glucose levels will be measured at baseline and at weeks 4, 8, 12, 16, 20, 24, 32 and 40. A HgbA1C will be used to measure each subject’s long-term glucose control and will be obtained at baseline and at weeks 8, 16 and 40.

## TENTATIVE SEQUENCE FOR INVESTIGATION

***Baseline*                        *Week #***

Test/event     (-4 to 0)  4      8   12     16    20 24     32     40 43

Physical exam        x      x          x     x           x     x x        x      x x

Anthropometrics       x               x                x        x x

CT abdomen            x                                                               x

OGTT                                                                x

IST                x                                                                x

IGF 1                 x                x x                x

LFTs                 x             x                      x         x x       x

Glucose               x    x     x        x        x       x     x      x x

Hgb A1C               x             x                       x          x

H. **POTENTIAL EXPERIMENTAL DIFFICULTIES AND ALTERNATIVE APPROACHES**

1. **Drug side effects**

*Recombinant human growth hormone (rhGH):* Side effects of rhGH include arthralgias and carpal tunnel symptoms (pain and paresthesias in the anterior wrist, palm and digits). These side effects are generally mild

and disappear either spontaneously or with rhGH dose reduction. Fluid retention may also occur in GHD patients and, less commonly, in non-GHD patients. In GHD patients, this is due to a dose-dependent

normalization of tissue hydration with GHD replacement (35). In non-GHD patients, fluid retention is generally mild and resolves spontaneously or with dose reduction. *Table 2* lists side effects attributed to rhGH in GH-deficient adults according to KIMS, a large pharmacoepidemiological survey(36):

Table 2 (from GH Replacement in Adults: the first 5 years of KIMS. Oxford PharmaGenesisTM Ltd:182-189, 2000):


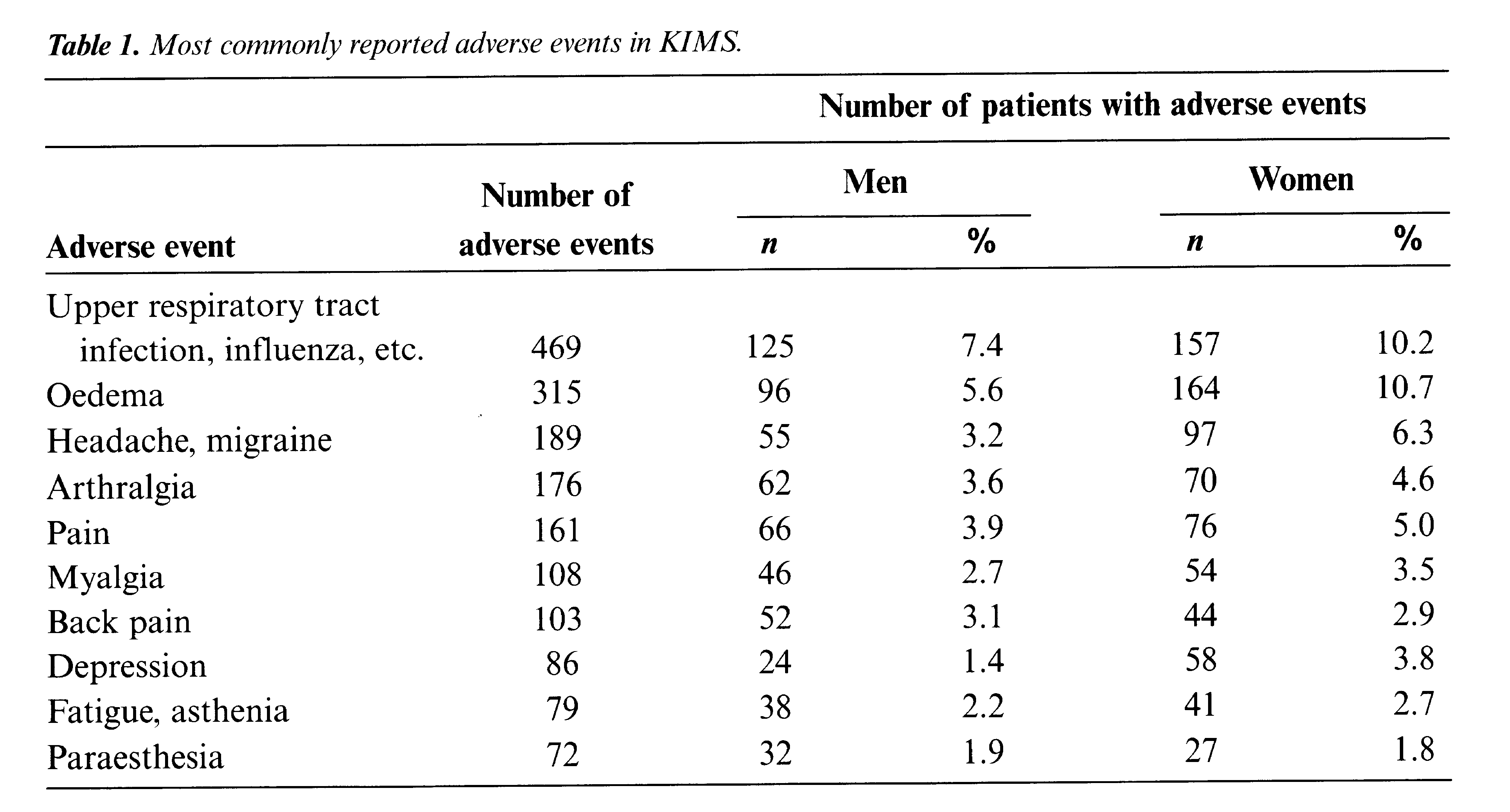


Serum glucose levels may transiently rise with rhGH due to its insulin antagonizing effects. It is expected that subjects in the combined pioglitazone/rhGH treatment group will be “protected” from this effect, whereas rhGH subjects *not* co-treated with a TZD may be susceptible. However, previous studies have shown that this susceptibility is reduced when moderate, rather than excessive, doses of rhGH are used (35). Marcus et. al showed that subjects with impaired glucose tolerance treated for 1 week with rhGH doses comparable to ours maintained similar glucose concentrations before and after treatment (37). In addition, Johannsson et. al showed that glycemic deterioration did not occur when rhGH doses similar to ours were used in people with underlying insulin resistance (25).

     The following measurements obtained on at least two separate occasions will be considered abnormal: (a) IGF-1 level greater than 2 standard deviations above the adult reference mean; OR (b) fasting glucose >126 mg/dl or random glucose > 200 mg/dl *plus* symptoms (ie: polyuria, polydipsia, ketosis) according to the revised ADA definition of diabetes mellitus. These abnormalities will result in the changes/interventions listed in *APPENDIX A* (please see *Page 13)*. *In order to maintain the study blind, a third-party investigator will be responsible for all drug dose adjustments during this study*.

The development of mild-to-moderate peripheral edema or symptoms indicative of rhGH-induced fluid retention (such as carpal tunnel syndrome, arthralgias, paresthesias) will also result in rhGH and/or TZD dose adjustments according to the algorithm in *APPENDIX B* (please see *Page 14*). In addition, the development of very severe or clinically life-threatening edema (ie: frank pulmonary edema, congestive heart failure) at any time during the study will result in prompt subject discontinuation.

*Thiazolidinediones (TZDs)*: TZDs can elevate liver function tests such as SGOT or SGPT. However, this is rarely clinically significant. Subjects will be discontinued from the study if LFTs are more than 2.5 times the upper normal range. In uncontrolled diabetics, weight gain may occur with TZDs as glucose levels fall. When serum glucose falls, glycosuria or excess glucose spillage in the urine ceases, and glucose is taken up into tissues. This often results in weight gain. Unlike uncontrolled diabetics, significant glycosuria does not usually

occur in IGT patients. Therefore, TZD-induced weight gain is expected to be minimal in our study. In addition, TZDs can infrequently cause peripheral edema that is usually mild. *Table 3* summarizes the side effects that may occur with TZDs, according to Foyt, et. al, 2000 (38,39,40).

Table 3 (Adapted from Thiazolidinediones. Diabetes Mellitus: a Fundamental and Clinical Text. Lippincott Williams and Wilkins:788-797, 2000):

1. LFT increase ♦ 0.2% incidence of ALT > 3X upper limit of normal (placebo

= 0.2%)

2. Anemia ♦ Hemoglobin decreases 0.64 g/dL; hematocrit decreases 2%

3. Edema ♦ 4.8% vs. placebo = 1.3%

4. Cardiac function ♦ No change in LV mass or function; no data on stroke volume

5. Weight gain ♦ 1.2 kg with 4 mg dose; 3.5 kg with 8 mg dose; 2.3 kg with 8

(mean) mg dose + metformin; not reported for 4 mg dose + sulfonylurea

Subjects will be weighed at each P.E. visit and examined very carefully for peripheral edema. The development of mild-to-moderate peripheral edema during the treatment period will result in pioglitazone and/or rhGH dose adjustments according to the algorithm in *APPENDIX A* (please see pages 13 and 14). In addition, the development of very severe or clinically life-threatening edema (ie: frank pulmonary edema, congestive heart failure) at any time during the study will result in prompt subject discontinuation.

    Subjects will be instructed to report side effects that develop during the study by calling the Clinical Studies Unit or by appearing for an unscheduled evaluation.

2. **Alternative Approaches**

Estrogen-treated subjects: Oral estrogen can sometimes antagonize the action of GH at the GH receptor level. This results in lower IGF 1 levels despite normal or even high levels of GH (41,42,43). Adult GHD women taking oral estrogen can require twice as much rhGH as adult GHD men because IGF 1 levels may be 50% lower in the estrogen-treated women.

      An alternative approach is to use a higher rhGH dose in estrogen-treated subjects. However, subjects in this study are non-GHD adults, and higher doses of rhGH may produce unacceptable side effects and abnormally high IGF 1 levels. Therefore, subjects will receive the same starting dose and groups will be matched for menopausal status and estrogen use.

TZDs versus diet plus exercise: An alternative approach that might obviate the need for insulin sensitizer therapy is diet and exercise. However, this approach will not be used in this study for two reasons. First, many

viscerally obese, IGT adults have already tried diet and exercise and failed. Secondly, previous studies have shown that diet and exercise cannot overcome the transient insulin resistance and hyperinsulinemia associated with rhGH (44,45).

E. **METHODS OF DATA ANALYSIS**

   Data will be analyzed using StatView software for Microsoft Windows (SAS Institute Inc., Cary, NC). All data will be expressed as the mean +/- SE. A Student’s *t* test will be used to compare baseline values of the subject groups. For measurements pertaining to visceral fat, insulin sensitivity and glucose metabolism, ANOVAs for repeated measures will be used to analyze differences between data obtained at all time points. Area under the curve (AUC) for glucose data will be determined using the trapezoid method. In addition, results will be considered significant at *P* < 0.05.

F. **SIGNIFICANCE**

A. **Prevention of type 2 diabetes** **in viscerally obese adults with IGT**

The growing health care burden of type 2 diabetes mellitus in the U.S. demands that newer, more effective diabetes prevention strategies be implemented. One way to achieve diabetes prevention is to target adults who are at greatest risk for developing diabetes, such as viscerally obese adults with IGT.

Recent studies like the Diabetes Prevention Program (DPP) Trial indicate that, in individuals with IGT, interventions that result in visceral fat reduction also significantly reduce the risk of developing overt diabetes. Unfortunately, in non-experimental settings, many obese individuals have had little success in achieving visceral fat reduction with current weight loss modalities such as diet, exercise or anorexogenic drugs. This lack of success is often related to noncompliance and recidivism with diet and exercise and with limited overall benefit, intolerable side effects and/or addictive potential with anorexogenic medications. Furthermore, the potent weight-reducing effects of bariatric surgery are often eclipsed by severe gastrointestinal complications postoperatively and, in rare circumstances, an increase in mortality.

Unlike these options, both pioglitazone and rhGH are generally well tolerated and are effective in promoting visceral fat loss. Furthermore, the combined use of both drugs over a long-term period might have a greater (ie: additive or synergistic) effect in reducing visceral fat and improving insulin sensitivity compared with the use of either drug alone. It is anticipated that pioglitazone plus prolonged rhGH administration will help to prevent diabetes mellitus in viscerally obese adults with IGT, in part through visceral fat reduction. If this is prospectively shown to occur, then the long-term savings in health care dollars with diabetes prevention would offset the short-term cost of either pioglitazone or growth hormone.

B. **Prevention of rhGH-induced insulin resistance during the first 6 weeks of therapy**

Many individuals who might otherwise benefit from the lipolytic and insulin sensitizing effects of rhGH go untreated because they have underlying (ie: pre-existing) insulin resistance. This includes the following patient populations:

• Viscerally obese adults with IGT

• Insulin-resistant adults with growth hormone deficiency (GHD)

• Adults with hereditary or acquired lipodystrophy syndromes (ie: HIV (+) patients on protease inhibitors)

• Women with PCOS and insulin resistance

• Viscerally obese adults with type 2 diabetes and a normal BMI (ie: people from the Indian subcontinent)

RhGH treatment is often withheld in these groups because of concerns about short-term adverse effects on insulin action and glucose metabolism. However, the combination of a TZD and rhGH is expected to prevent or minimize any short-term worsening in insulin resistance in such individuals and thus allow treatment to continue. Therefore, the aforementioned groups are potential candidates for this combination therapy in the future.

C. **Improvement in Insulin Sensitivity in Adults with Growth Hormone Deficiency (GHD)**

RhGH is FDA-approved for replacement in adults with documented growth hormone deficiency, or GHD. By definition, GHD adults have inadequate growth hormone production and secretion due to pituitary/hypothalamic injury (ie: from a large tumor, prior radiation treatment or brain trauma). GHD adults typically have an abnormal body composition, including diminished lean body mass and increased body fat. In addition, death due to cardiovascular disease is common in this population, and it is believed that both visceral obesity and insulin resistance contribute to the increase in cardiovascular mortality. Compared with the use of rhGH alone, it is possible that rhGH plus pioglitazone might help to optimize body composition and insulin sensitivity in GHD adults and, therefore, reduce their cardiovascular risk. It is also possible that the frequent untoward occurrence of transient insulin resistance during the first 6 weeks of rhGH replacement in GHD adults might be prevented by co-treatment with pioglitazone.

D. **Reduction in CV Disease Risk Associated with Visceral Obesity and Insulin Resistance**

Loss of visceral fat helps to reduce cardiovascular (CV) risk indirectly by improving the metabolic abnormalities associated with visceral fat excess: dyslipidemia, hypertension, insulin resistance and hyperglycemia. TZDs and rhGH have been independently shown to effectively reduce visceral fat in insulin-

resistant, abdominally obese adults and ameliorate metabolic variables such as hyperlipidemia and insulin resistance that are associated with visceral adiposity. It is possible that the combined use of a TZD with rhGH will result in an even greater reduction in visceral fat and insulin resistance compared with the use of either drug alone, and this in turn might lead to a reduction in cardiovascular morbidity and mortality.

While rhGH and TZDs share in common the ability to reduce visceral fat and increase insulin sensitivity, each drug also acts in a different manner to potentially improve cardiovascular outcome. In viscerally obese growth hormone deficient (GHD) adults, for example, rhGH has been shown to significantly reduce carotid artery intima-media thickness and improve brachial artery blood flow. After 3-6 months of rhGH administration, the carotid artery intima-media thickness of adult GHD men as measured by ultrasound becomes very similar to that of healthy age- and sex-matched controls (46). These data suggest a possible therapeutic role for rhGH in reducing long-term cardiovascular disease and stroke risk by *directly* improving vascular morphology and function. Similarly, TZDs have been shown to yield a multitude of benefits on cardiovascular risk factors that might be unrelated to changes in insulin sensitivity or glucose metabolism. These include improvements in cardiovascular markers such as C-reactive protein (CRP), interleukin-6, and PAI-1, and a reduction in fibrinogen levels, platelet aggregation and dyslipidemia (27). In addition, TZDs may have direct cardiovascular benefits that include decreasing intima-media thickness, reducing neovascularization after coronary vessel injury and improving arterial blood flow (27). Therefore, both TZDs and rhGH appear to have direct and indirect beneficial long-term effects on the cardiovascular system, and many of these benefits are mediated by a reduction in visceral fat and an improvement in insulin sensitivity. It is expected that the long-term combined use of rhGH and pioglitazone in viscerally obese, insulin resistant adults will help to reduce cardiovascular risk factors and cardiovascular disease.

G. **REFERENCES *(for Protocol only)***

1. Rexrode KM, Carey VJ, Hennekens CH, Walters EE, Colditz GA, Stampfer MJ, Willett WC, Manson JE. Abdominal adiposity and coronary heart disease in women. JAMA 280:1843-1848, 1998.
2. Montague CT, O’Rahilly S. The perils of portliness: causes and consequences of visceral adiposity. Diabetes 49:883-888, 2000.
3. Stone NJ, Kushner R. Effects of dietary modification and treatment of obesity: emphasis on improving vascular outcomes. Medical Clinics of North America 84:95-122, 2000.
4. Brochu M, Poehlman ET, Ades PA. Obesity, fat distribution, and coronary artery disease. J Cardiopulm Rehabil 20:96-108, 2000.
5. Busetto L. Visceral obesity and the metabolic syndrome: effects of weight loss. Nutr Metab Cardiovasc Dis 11:195-204, 2001.
6. Tochikubo O, Miyajima E, Okabe K, Imai K, Ishii M. Improvement of multiple coronary risk factors in obese hypertensives by reduction of intra-abdominal visceral fat. Jpn Heart J 35:715-25, 1994.
7. Metz JA, Stern JS, Kris-Etherton P, Reusser ME, Morris CD, Hatton DC, Oparil S, Haynes BR, Resnick LM, Pi-Sunyer FX, Clark S, Chester L, McMahon M, Snyder GW, McCarron DA. A randomized trial of improved weight loss with a prepared meal plan in overweight and obese patients: impact on cardiovascular risk reduction. Arch Intern Med 160:2150-2158, 2000.
8. Pouliot MC, Despres JP, Nadeau A, Moorjani S, Prudhomme D, Lupien PJ, Tremblay A, Bouchard C. Visceral obesity in men: associations with glucose tolerance, plasma insulin, and lipoprotein levels. Diabetes 41:826-834, 1992.
9. Meigs JB, Nathan DM, Wilson PW, Cupples AL, Singer DE. Metabolic risk factors worsen continuously across the spectrum nondiabetic glucose tolerance: The Framingham Offspring Study. Ann Intern Med 128:523-533, 1998.
10. Tominaga M, Eguchi H, Manaka H, Igarashi K, Kato T, Sekikawa A. Impaired glucose tolerance is a risk factor for cardiovascular disease, but not impaired fasting glucose. Diabetes Care 22:920-924, 1999.
11. Rodriguez BL, Curb JD, Burchfiel CM, Huang B, Sharp DS, Ye Lu G, Fujimoto W, Yano K. Impaired glucose tolerance, diabetes and cardiovascular disease risk factor profiles in the elderly: The Honolulu Heart Program. Diabetes Care 19:587-590, 1996.
12. Lefebvre PJ, Scheen AJ. The postprandial state and risk of cardiovascular disease. Diabet Med 15:S63-S68, 1998.
13. Rice B, Janssen I, Hudson R, Ross R. Effects of aerobic or resistance exercise and/or diet on glucose tolerance and plasma insulin levels in obese men. Diabetes Care 22:684-691, 1999.
14. Katzel LI, Bleecker ER, Colman EG, Rogus EM, Sorkin JD, Goldberg AP. Effects of weight loss vs aerobic exercise training on risk factors for coronary artery disease in healthy, obese, middle-aged and older men. A randomized controlled trial. JAMA 274:1915-1921, 1995.
15. Sonnichsen AC, Richter WO, Schwandt P. Benefit from hypocaloric diet in obese men depends on the extent of weight-loss regarding cholesterol, and on a simultaneous change in body fat distribution regarding insulin sensitivity and glucose tolerance. Metabolism 41:1035-1039, 1992.
16. Muscelli E, Camastra S, Catalano C, Galvan AQ, Ciociaro D, Baldi S, Ferrannini E. Metabolic and cardiovascular assessment in moderate obesity: effect of weight loss. J Clin Endocrinol Metab 82:2937-2943, 1997.
17. Dengel DR, Pratley RE, Hagberg JM, Rogus EM, Goldberg AP. Distinct effects of aerobic exercise training and weight loss on glucose homeostasis in obese sedentary men. Journal of Applied Physiology 81:318-325, 1996.
18. Smutok MA, Reece C, Kokkinos PF, Farmer C, Dawson P, Shulman R, DeVane-Bell J, Patterson J, Charabogos C, Goldberg AP. Aerobic versus strength training for risk factor intervention in middle-aged men at high risk for coronary heart disease. Metabolism: Clinical and Experimental 42:177-184,1993.
19. Despres JP, Pouliot MC, Moorjani S, Nadeau A, Tremblay A, Lupien PJ, Theriault G, Bouchard C. Loss of abdominal fat and metabolic response to exercise training in obese women. American Journal of Physiology 261:E159-167, 1991.
20. Fujioka S, Matsuzawa Y, Tokunaga K, Kawamoto T, Kobatake T, Keno Y, Kotani K, Yoshida S, Tarui S. Improvement in glucose and lipid metabolism associated with selective reduction of intra-abdominal visceral fat in premenopausal women with visceral fat obesity. International Journal of Obesity 15:853-859, 1991.
21. Eriksson KF, Lindgarde F. Prevention of type 2 (non-insulin dependent) diabetes mellitus by diet and physical exercise. The 6-year Malmo feasibility study. Diabetologia 34:891-898, 1991.
22. Diabetes Prevention Program Research Group. Reduction in the incidence of type 2 diabetes with lifestyle

intervention or metformin. NEJM 346:393-403, 2002.

23. Drake WM, Howell SJ, Monson JP, Shalet SM. Optimizing GH therapy in adults and children. Endocrine Reviews

22:425-450, 2001.

1. Clasey JL, Weltman A, Patrie J, Weltman JY, Pezzoli S, Bouchard C, Thorner MO, Hartman ML. Abdominal visceral fat and fasting insulin are important predictors of 24-hour GH release independent of age, gender, and other physiological factors. J Clin Endocrinol Metab 86:3845-3852, 2001.
2. Johannsson G, Per M, Lars L, Malin O, Kaj S, Per B, Lars S, Bengt-Ake B. Growth hormone treatment of abdominally obese men reduces abdominal fat mass, improves glucose and lipoprotein metabolism, and reduces diastolic blood pressure. J Clin Endocrinol Metab 82:727-734, 1997.
3. Hamm JK, el Jack AK, Pilch PF, Farmer SR. Role of PPAR gamma in regulating adipocyte differentiation and insulin-responsive glucose uptake. Ann N Y Acad Sci 892:134-145, 1999.
4. Parulkar A, Pendergrass ML, Granda-Ayala R, Lee TR, Fonseca VA. Nonhypoglycemic effects of thiazolidinediones. Ann Intern Med 134:61-71, 2001.
5. Olefsky JM. Treatment of insulin resistance with peroxisome proliferator-activated receptor (gamma) agonists. J Clin Invest 106:467-472, 2000.
6. Sugimoto M, Takeda N, Nakashima K, Okumura S, Takami K, Yoshino K, Hattori J, Ishimori M, Takami R, Sasaki A, Yasuda K. Effects of troglitazone on hepatic and peripheral insulin resistance induced by growth hormone excess in rats. Metabolism 47:783-787, 1998.

29a. Miyazaki Y, Mahankali A, Matsuda M, Hardies J, Cusi K, Mardarino LJ, Defronzo RA. Effect of pioglitazone on

abdominal fat distribution and insulin sensitivity in type 2 diabetic patients. J Clin Endocrinol Metab 87:2784-2791.

1. Han TS, McNeill G, Seidell JC, Lean ME. Predicting intra-abdominal fatness from anthropometric measures: the influence of stature. Int J Obes Relat Metab Disord 21:587-593, 1997.
2. Raikkonen K, Matthews KA, Kuller LH. Anthropometric and psychosocial determinants of visceral obesity in healthy postmenopausal women. Int J Obes Relat Metab Disord 23:775-782, 1999.
3. van der Kooy K, Seidell JC. Techniques for the measurement of visceral fat: a practical guide. Int J Obes Relat Metab Disord 17:187-196, 1993.
4. Reaven GM, Brand RJ, Chen Y-DI, et al. 1993 Insulin resistance and insulin secretion are determinants of oral glucose tolerance in normal individuals 42:1324-1322, Diabetes.
5. Pei D, Jones CNO, Bhargava R, Chen Y-DI, Reaven GM. 1994 Evaluation of octreotide to assess insulin-mediated glucose disposal by the insulin suppression test 37:843-845, Diabetologia.
6. Consensus. Critical evaluation of the safety of recombinant human growth hormone administration: statement from the growth hormone research society. J Clin Endocrinol Metab 86:1868-1870, 2001.
7. Wilton P, Koppeschaar HPF. Safety of growth hormone replacement in adults. GH Replacement in Adults: the first 5 years of KIMS. Oxford PharmaGenesisTM Ltd:182-189, 2000.
8. Marcus R, Butterfield G, Holloway L, Gilliland L, Baylink DJ, Hintz RL, Sherman BM. Effects of short term administration of recombinant human growth hormone to elderly people. J Clin Endocrinol Metab 70:519-527, 1990.
9. Foyt HL, Ghazzi MN, Hanley RM, Saltiel AR, Whitomb RW. Thiazolidinediones. Diabetes Mellitus: a Fundamental and Clinical Text. Lippincott Williams and Wilkins:788-797, 2000.
10. Rosiglitazone abstracts. Diabetes 48:Suppl 1, 1999.
11. Package insert, pioglitazone hydrochloride. 2005 Actos Takeda Pharmaceuticals America, Lincolnshire,

IL.

1. Holloway L, Butterfield G, Hintz RL, Gesundheit N, Marcus R. Effects of recombinant human growth hormone on metabolic indices, body composition, and bone turnover in healthy elderly women. J Clin Endocrinol Metab 79:470-479, 1994.
2. Span JP, Pieters GF, Sweep CG, Hermus AR, Smals AG. Gender difference in insulin-like growth factor 1 response to growth hormone (GH) treatment in GH-deficient adults: role of sex hormone replacement. J Clin Endocrinol Metab 85:1121-1125, 2000.
3. Cook DM, Ludlam WH, Cook MB. Route of estrogen administration helps to determine growth hormone (GH) replacement dose in GH-deficient adults. J Clin Endocrinol Metab 84:3956-3960, 1999.
4. Snyder DK, Underwood LE, Clemmons DR. Persistent Lipolytic effect of exogenous growth hormone during caloric restriction. The American Journal of Medicine 98:129-134, 1995.
5. Zachwieja JJ, Toffolo G, Cobelli C, Bier DM, Yarasheski KE. Resistance exercise and growth hormone administration in older men: effects on insulin sensitivity and secretion during a stable-label intravenous glucose tolerance test. Metabolism 45:254-60, 1996.
6. Pfeifer M, Verhovec R, Zizek B, Prezelj J, Poredos P, Clayton RN. Growth hormone (GH) treatment reverses early atherosclerotic changes in GH-deficient adults. JCEM 84:453-457, 1999.

# APPENDIX B: RhGH Dose Adjustment for IGF-1 > 2 SD Above Lab Reference Mean

1. **For subjects receiving *RhGH* 6-8 mcg/kg/day**, reduce *RhGH* dose by 2 mcg/kg/day. Repeat IGF-1 in 4 weeks. If IGF-1 level is within normal range, continue current *RhGH* dose. If IGF-1 level is still elevated

(>2 SD), go back to *APPENDIX B*.

B. **For subjects already dose-reduced to *RhGH* 4 mcg/kg/day**, discontinue from study.
